# Supplementary material for: Novel Variant in PLAG1 in a Familial Case with Silver–Russell Syndrome Suspicion
Source: Genes (Basel). 2020 Dec 5;11(12):1461. doi: 10.3390/genes11121461 (PMC7762056; doi:10.3390/genes11121461)
Supplement: Supplementary file 1 [file genes-11-01461-s001.zip › genes-1005660/LEGEND SUPPLEMENTARY TABLE 1.docx]

**LEGEND SUPPLEMENTARY TABLE 1**

**Supplementary table S1**: molecular and clinical findings for patients with *PLAG1* gene alterations, in current study and described in the bibliography.

**Supplementary table S2**: molecular and clinical findings for patients with *CDKN1C* gene alterations, described in the bibliography.

**Supplementary table S3**: molecular and clinical findings for patients with *HMGA2* gene alterations or rearrangements involving this gene, described in the bibliography

**Supplementary table S4**: molecular and clinical findings for patients with *IGF2* gene, described in the bibliography.

Abbreviations: +: presence of the specified clinical finding; -: absence of the specified clinical finding; ND: no data for the specified clinical finding; NA: not applicable; y: years old.

Comments: these tables were adapted from Masunaga et al. [15].
